# Developing diagnostic tools for canine periodontitis: combining molecular techniques and machine learning models

**DOI:** 10.1186/s12917-023-03668-3

**Published:** 2023-09-18

**Authors:** Avika Ruparell, Matthew Gibbs, Alison Colyer, Corrin Wallis, Stephen Harris, Lucy J. Holcombe

**Affiliations:** Waltham Petcare Science Institute, Melton Mowbray, Leicestershire, UK

**Keywords:** Canine, Periodontal disease, Periodontitis, Diagnosis, Quantitative polymerase chain reaction, qPCR, Microbiome, Microbiota, Biomarkers

## Abstract

**Background:**

Dental plaque microbes play a key role in the development of periodontal disease. Numerous high-throughput sequencing studies have generated understanding of the bacterial species associated with both canine periodontal health and disease. Opportunities therefore exist to utilise these bacterial biomarkers to improve disease diagnosis in conscious-based veterinary oral health checks. Here, we demonstrate that molecular techniques, specifically quantitative polymerase chain reaction (qPCR) can be utilised for the detection of microbial biomarkers associated with canine periodontal health and disease.

**Results:**

Over 40 qPCR assays targeting single microbial species associated with canine periodontal health, gingivitis and early periodontitis were developed and validated. These were used to quantify levels of the respective taxa in canine subgingival plaque samples collected across periodontal health (PD0), gingivitis (PD1) and early periodontitis (PD2). When qPCR outputs were compared to the corresponding high-throughput sequencing data there were strong correlations, including a periodontal health associated taxa, *Capnocytophaga* sp. COT-339 (*r*_*s*_ =0.805), and two periodontal disease associated taxa, Peptostreptococcaceae XI [G-4] sp. COT-019 (*r*_*s*_=0.902) and Clostridiales sp. COT-028 (*r*_*s*_=0.802). The best performing models, from five machine learning approaches applied to the qPCR data for these taxa, estimated 85.7% sensitivity and 27.5% specificity for *Capnocytophaga* sp. COT-339, 74.3% sensitivity and 67.5% specificity for Peptostreptococcaceae XI [G-4] sp. COT-019, and 60.0% sensitivity and 80.0% specificity for Clostridiales sp. COT-028.

**Conclusions:**

A qPCR-based approach is an accurate, sensitive, and cost-effective method for detection of microbial biomarkers associated with periodontal health and disease. Taken together, the correlation between qPCR and high-throughput sequencing outputs, and early accuracy insights, indicate the strategy offers a prospective route to the development of diagnostic tools for canine periodontal disease.

**Supplementary Information:**

The online version contains supplementary material available at 10.1186/s12917-023-03668-3.

## Background

Microbes play a fundamental role in the aetiology of periodontal disease development; their integration into plaque biofilms and subsequent, progressive accumulation are key in the activation of the host inflammatory response [1–4]. As such, a number of scientific investigations have been conducted to understand the complexity of the canine oral microbiome. Characterisation of the microbiota has elucidated that only 16.4% of taxa are shared with the human oral cavity [5]. As a consequence, recent exploration into canine periodontal disease has focused on determining the associations of microbial taxa with periodontal health or disease, using high throughput sequencing (HTS) on samples collected from cross-sectional and longitudinal surveys spanning subsets of the clinical spectrum [6–11].

Fundamental insights into potential microbial biomarkers of periodontal health and disease deliver an opportunity to improve diagnosis of the disease in dogs. Clinical accounts vary in the prevalence rates reported, suggesting between 44% and 100% of dogs are affected [1, 12–15]. However, only 9–20% of dogs are diagnosed in first opinion practice due to the nature of examinations predominantly being based on conscious visual assessments [16–18]. Full characterisation of the extent of periodontal disease requires general anaesthesia, through which the level of clinical attachment loss and bone loss can be accurately determined using periodontal probing and intra-oral radiographs [19].

Development of the disease follows a phased, graduated progression; initiating with inflamed gingiva (gingivitis), which can subsequently progress to periodontitis where the alveolar bone and periodontal ligaments are destroyed [18, 20]. Advancement into the latter phases not only increases the risk of irreversible, localised consequences such as potential for abscesses, ulcers and tooth loss, but links to possible repercussions in systemic health [21–26]. Dental discomfort in dogs may also be coupled with physical and behavioural changes; examples include eating abnormalities, associated weight loss and signs of distress [18, 20].

Under-diagnosis in general veterinary clinics and the potential severity of the disease amplifies the requirement for practical and efficacious tools in the diagnostics arena; here we outline an approach for canine periodontal disease. Based on consolidation of clinical insights developed from a number of cross-sectional and longitudinal HTS studies [6, 7, 9, 11], a portfolio of qPCR assays was developed to enable quantification of specific single species of bacteria. Assays selected for development were based on statistical significance (*p* < 0.05), prevalence (number of samples positive for bacterial species in periodontal health verses periodontal disease), abundance (≥0.05%) and fold-change (> 2-fold). We then utilised some of these assays to pursue our main objective, to demonstrate quantitative polymerase chain reaction (qPCR) as an appropriate method for the detection of microbial biomarkers indicative of periodontal health and disease, by comparing outputs of qPCR and HTS applied to the same sample set. As a molecular tool, qPCR offers a targeted, rapid, and relatively cost-effective method for quantifying specific bacterial species compared to other approaches, such as HTS, which profile entire communities. Kwon et al. [27] recently applied qPCR to quantify 11 human periodontopathic species from canine plaque samples, and suggested *Treponema denticola* as a prognostic biomarker for periodontitis in dogs. Here, we evaluate canine periodontal health and disease associated microbial species for their performance as diagnostic biomarkers, utilising five classification machine learning techniques to optimise sensitivity and specificity parameters. Overall, the employment of qPCR in the detection of a microbial biomarker associated with a specific clinical oral health status offers a potential strategy to improve diagnosis of periodontitis in dogs.

## Results

### Development and validation of single species qPCR assays

Forty-one qPCR assays each targeting a single bacterial species were successfully developed and validated (Table 1). The robustness of each qPCR assay was evaluated via efficiency and specificity parameters. Efficiency, a measure of the percentage of target molecules that are copied per PCR cycle, was determined as ≥90% for 32 of the 41 assays (78%) (Table 1). The remaining nine assays (22%) indicated efficiencies between 79.21% and 89.54% (Table 1). The efficiency of *Moraxella* sp. COT-017, 79.21%, was accepted given its close proximity to the cut-off (≥80%). Specificity of each assay’s target detection was established by screening a library comprising 415 clones, representing different bacterial species of canine oral microbiota [5]. All 41 assays conformed to the threshold criteria, confirming that cross reactivity was sufficiently low to be considered negligible.

**Table 1:** Full and truncated quantitative polymerase chain reaction (qPCR) assay names and efficiency data

Full Assay Name	Truncated Assay Name	Assay Efficiency (%)	Limit of quantification (LOQ)
*Actinomyces* sp. COT-083	COT-083	92.42	34.00
*Actinomyces* sp. COT-252	COT-252	90.73	35.00
*Anaerovorax* sp. COT-125	COT-125	99.96	35.57
*Bacteroides* sp. COT-040	COT-040	85.76	36.94
*Bergeyella zoohelcum* COT-186	COT-186	97.51	35.47
*Capnocytophaga* sp. COT-339	COT-339	85.86	35.00
Clostridiales sp. COT-005	COT-005	95.37	35.30
Clostridiales sp. COT-028	COT-028	83.21	36.00
*Desulfomicrobium orale* COT-008	COT-008	95.56	35.20
*Filifactor* sp. COT-064	COT-064	93.29	35.00
*Frigovirgula* sp. COT-007	COT-007	97.55	35.00
*Fusobacterium* sp. COT-169	COT-169	90.18	34.00
*Fusobacterium* sp. COT-189	COT-189	93.22	33.00
*Gemella palaticanis* COT-089	COT-089	89.27	36.00
*Granulicatella* sp. COT-095	COT-095	97.04	36.00
*Helcococcus* sp. COT-069	COT-069	95.79	36.73
Lachnospiraceae XIVa [G-4] sp. COT-099	COT-099	97.79	33.90
Lachnospiraceae XIVa [G-5] sp. COT-024	COT-024	90.05	32.00
*Leptotrichia* sp. COT-345	COT-345	85.43	35.00
*Moraxella* sp. COT-017	COT-017	79.21	36.00
*Moraxella* sp. COT-018	COT-018	94.32	34.00
*Neisseria animaloris* COT-016	COT-016	90.73	35.00
*Odoribacter denticanis* COT-084	COT-084	95.15	36.00
Pasteurellaceae sp. COT-271	COT-271	90.49	34.98
*Peptococcus* sp. COT-044	COT-044	91.92	35.00
Peptostreptococcaceae XI [G-1] sp. COT-004	COT-004	96.92	34.59
Peptostreptococcaceae XI [G-1] sp. COT-006	COT-006	97.43	33.40
Peptostreptococcaceae XI [G-3] sp. COT-104	COT-104	94.77	34.91
Peptostreptococcaceae XI [G-4] sp. COT-019	COT-019	95.79	36.00
Peptostreptococcaceae XI [G-6] sp. COT-068	COT-068	90.66	33.00
Peptostreptococcaceae XIII [G-1] sp. COT-030	COT-030	91.08	36.50
Peptostreptococcaceae XIII [G-2] sp. COT-077	COT-077	98.56	33.60
*Peptostreptococcus* sp. COT-033	COT-033	94.88	34.00
*Peptostreptococcus* sp. COT-227	COT-227	96.22	34.00
*Porphyromonas cangingivalis* COT-109	COT-109	99.29	35.00
*Porphyromonas gingivicanis* COT-022	COT-022	89.07	35.00
*Porphyromonas gulae* I COT-052	COT-052	89.54	34.00
*Porphyromonas macacae* COT-192	COT-192	97.51	34.73
*Porphyromonas* sp. COT-108	COT-108	94.96	34.25
Synergistales [F-2,G-1] sp. COT-138	COT-138	83.01	32.92
*Tannerella forsythus* COT-023	COT-023	95.64	34.00

Efficiency provides a measure of the percentage of target molecules that are copied per PCR cycle per qPCR assay. Limit of quantification (LOQ) represents the lowest level of input target sequence that can be accurately quantified

### HTS and qPCR outputs indicate good alignment, overall and for individual assays

**Fig. 1:**
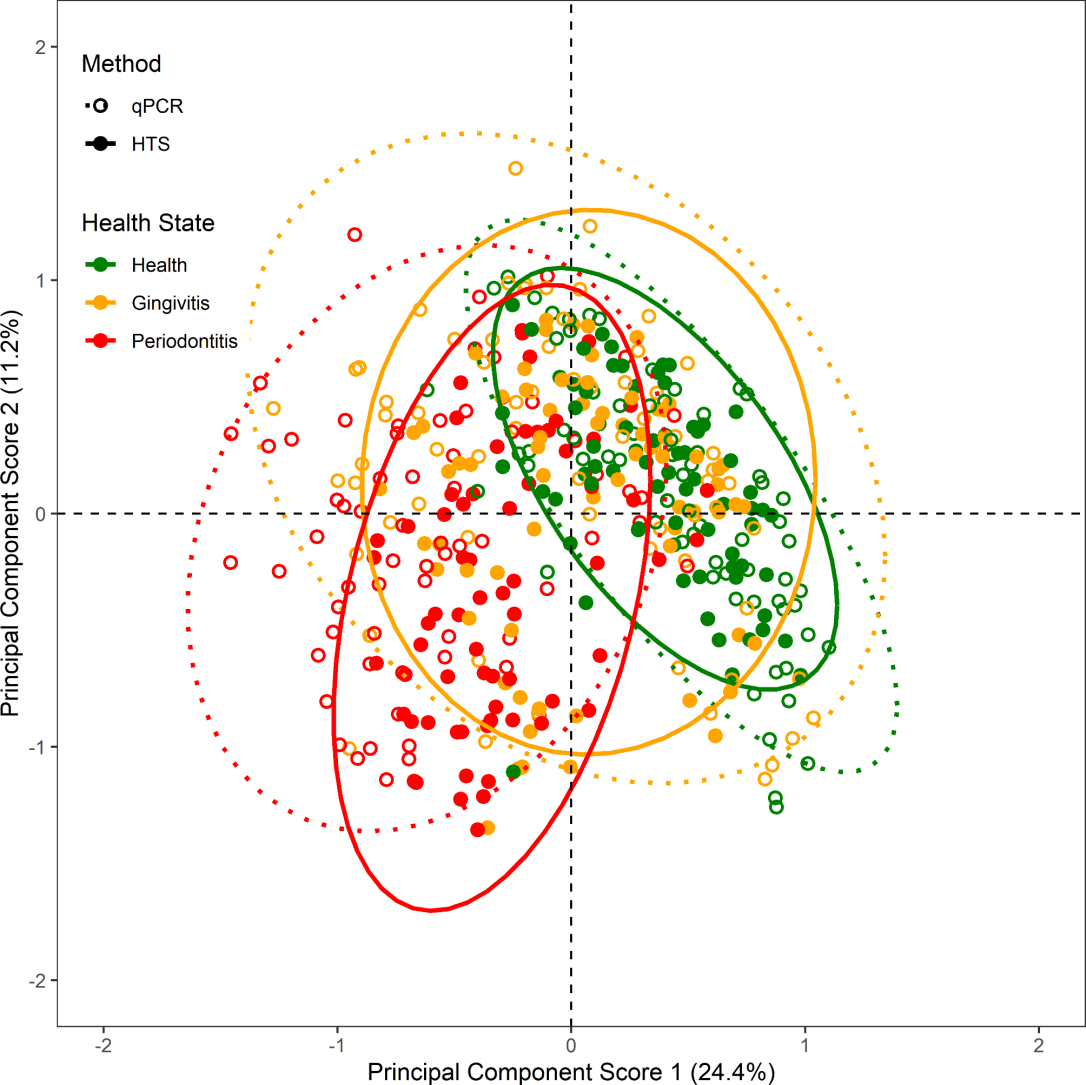
Principal component scores with ellipses representing 95% confidence regions from analysis performed on the counts and proportions identified via high-throughput sequencing (HTS) and quantitative polymerase chain reaction (qPCR) respectively. Discriminated by health state: health (green), gingivitis (orange) and periodontitis (red); and analytical method: qPCR (empty data points; dotted ellipse) and HTS (filled data points; solid ellipse)

To explore the potential of the selected set of taxa for practical application as microbial biomarkers, the relationships between the qPCR assay outputs and the corresponding HTS outputs following analysis of the suite of plaque samples was examined. PCA was used to explore potential variability between the two analysis methods; the first component explained 24.4% and the second component 11.2% of the variability in the qPCR and HTS data (Fig. 1). Discrete clustering was observed by health state with the health and periodontitis samples forming discrete groups in PC1 although there was some overlap. The gingivitis samples were most variable, with the samples dispersed across both the health and periodontitis clusters. This was apparent irrespective of the technology; both were shown to be similar with no distinctive grouping.

**Fig. 2:**
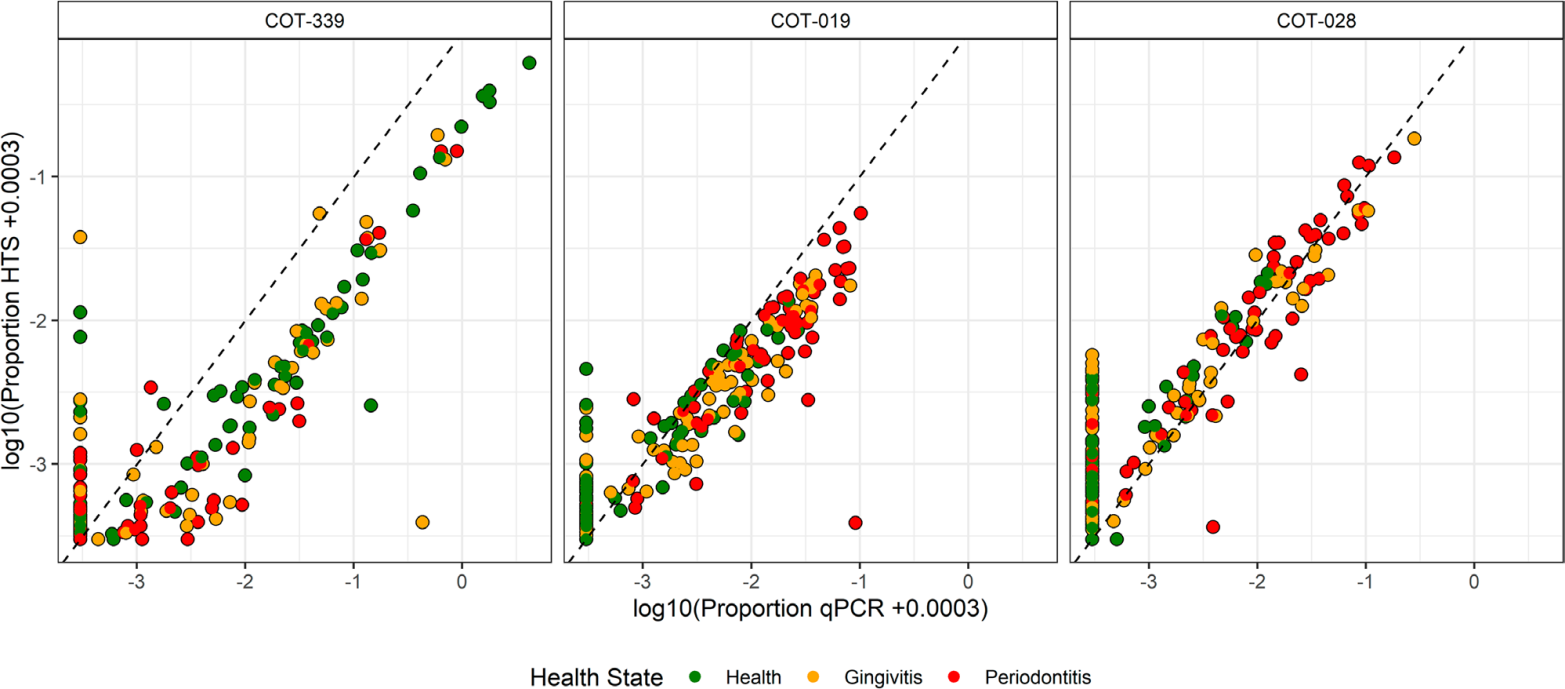
Correlations between high-throughput sequencing (HTS) and quantitative polymerase chain reaction (qPCR) datasets acquired from analysis of the same sample cohort. *Capnocytophaga* sp. COT-339, Peptostreptococcaceae XI [G-4] sp. COT-019 and Clostridiales sp. COT-028. Samples are discriminated by periodontal health state: health (green), gingivitis (orange) and periodontitis (red)

To enable species specific insights, the qPCR data from each assay was plotted against the HTS data from Davis et al. [6] for each corresponding plaque sample. Of the 41 qPCR assays, 30 were strongly correlated (*r* > 0.8) and 10 were moderately correlated (0.8 > *r* > 0.5) according to Pearson’s correlations (Supplementary Table 1). According to Spearman’s Rank correlations, 25 of the 41 qPCR assays were strongly correlated (*r*_*s*_ > 0.8) and 13 were moderately correlated (0.8 > *r*_*s*_ > 0.5) (Supplementary Table 1). Examples of strongly correlating assays include the periodontal health associated taxa, *Capnocytophaga* sp. COT-339, and periodontal disease associated taxa, Peptostreptococcaceae XI [G-4] sp. COT-019 and Clostridiales sp. COT-028. For these species, the HTS and qPCR assay technologies were strongly correlated using both Pearson’s (*r* = 0.874, *r* = 0.905 and *r* = 0.902 respectively, *p* < 0.001) (Supplementary Table 1) and Spearman’s rank (*r*_*s*_ =0.805, *r*_*s*_ =0.902 and *r*_*s*_ =0.802 respectively, *p* < 0.001) methods (Fig. 2). For these three assays, reaction efficiencies of 85.86%, 95.79% and 83.21%, respectively, were determined (Table 1). It is noteworthy that a number of plaque samples indicated zero relative abundance (i.e. below limit of assay detection) with the species specific qPCR probes, but the taxa were detected using HTS.

### Canine periodontal disease associated species differ in their diagnostic potential

**Fig. 3:**
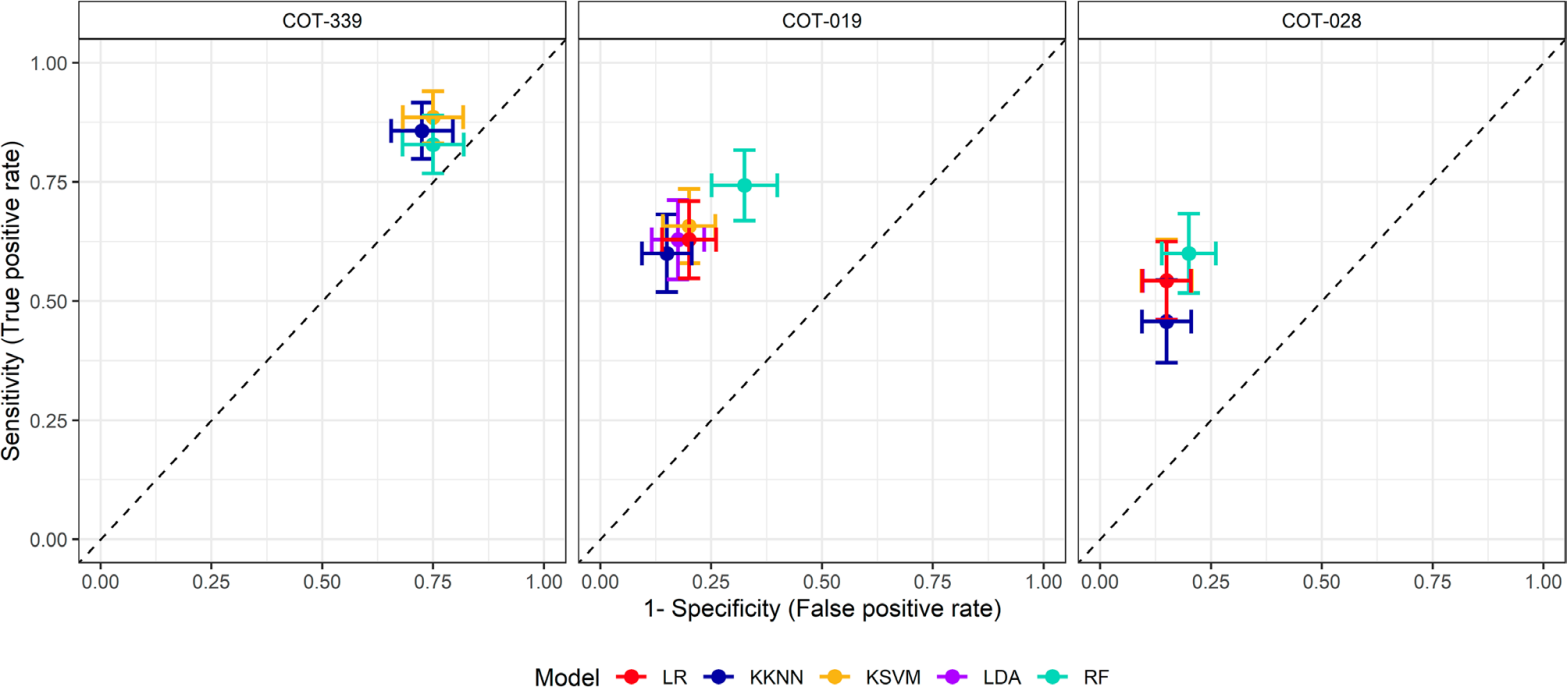
Sensitivity against 1- Specificity estimations using 5 machine learning classification models, for Peptostreptococcaceae XI [G-4] sp. COT-019, Clostridiales sp. COT-028 and *Capnocytophaga* sp. COT-339. Methods employed were logistic regression (LR, red), weighted k-nearest neighbour (KKNN, dark blue), kernel support vector machines (KSVM, yellow), linear discriminant analysis (LDA, purple) and random forest (RF, aqua). Average estimates +/- standard deviation are presented from the bootstrap testing of the optimised trained models

The plaque sample qPCR outputs from assays developed against *Capnocytophaga* sp. COT-339, Peptostreptococcaceae XI [G-4] sp. COT-019 and Clostridiales sp. COT-028 were modelled to assess their sensitivity (correct classification of periodontitis samples) and specificity (correct classification of non-periodontitis samples) (Fig. 3). For *Capnocytophaga* sp. COT-339, three models gave outputs with estimated 82.9–88.6% sensitivity and 25.0-27.5% specificity, with the KSVM approach providing best perfoming model with 85.7% (5.9%) sensitivity and 27.5% (7.0%) specificity. For Peptostreptococcaceae XI [G-4] sp. COT-019, the outputs of all five models estimated 60.0-74.3% sensitivity and 67.5–85.0% specificity with the random forest approach providing the best performing model with 74.3% (7.4%) sensitivity and 67.5% (7.4%) specificity. For Clostridiales sp. COT-028, all five models gave outputs and these estimated 45.7–60.0% sensitivity 80.0–85.0% specificity with the random forest approach providing the best performing model with 60.0% (8.4%) sensitivity and 80.0% (6.1%) specificity.

## Discussion

Comprehensive evaluations of the oral plaque microbiota in canine periodontal disease have enabled understanding of the associations of specific bacterial taxa with periodontal health and disease in dogs. Based on these invaluable insights, we have established a portfolio of more than 40 qPCR assays which selectively target single bacterial species, enabling their relative levels to be accurately quantified from a given plaque DNA sample. Building the panel of assays complements the knowledge of periodontal health status associations with the addition of rigorously validated molecular tools, opening up opportunities for diagnostics to be developed.

The performance of our portfolio of single-species targeted qPCR assays was assessed by comparing their bacterial detection capability against the equivalent findings delivered via a HTS approach. This indicated moderate to strong overall alignment between the targeted, qPCR approach and the broad-spectrum HTS technology. HTS targets ubiquitous 16S rDNA sequence, thereby promoting amplification of all the members of the microbial community within a given sample [28]. In contrast, the approach undertaken for qPCR assay design exploits novelty in regions of the 16S rRNA sequence to enable targeting of individual bacterial species [29]. The qPCR assays were able to discriminate subgingival plaque samples from dogs with healthy gingiva from those with periodontitis, where assessments were based on clinical diagnosis under general anaesthesia by a Diplomat of the European Veterinary Dental College. This result was similar to the observations from the HTS cross-sectional study which employed the same clinical plaque samples [6].

The subsequent evaluation of the two technologies focused on the individual qPCR assays and delivered varying correlation coefficients. Approximately 60% of the validated assays indicated a high correlation (*r*_*s*_ >0.8) with Spearman’s correlation method including those targeting the canine periodontal health associated taxa, *Capnocytophaga* sp. COT-339, and periodontal disease associated taxa, Peptostreptococcaceae XI [G-4] sp. COT-019 and Clostridiales sp. COT-028. These findings further reinforce the feasibility of using a molecular tool such as qPCR to detect microbial biomarkers of canine periodontal disease and provide quantifiable sample-to-sample discrimination comparable to a HTS approach. Scientific publications, additionally, show diversity in the correlation between the two technologies [30–32]. One investigation concluded substantial agreement (R^2^ = 0.872 and R^2^ = 0.929) between the methods for targeting cheese microbiota [30]. Another comparison, conducted to characterise vaginal lactobacilli reported mixed findings; proportions of one lactobacilli, *Lactobacillus crispatus*, were well correlated (*r* = 0.79, *p* < 0.001), while that of another, *Lactobacillus iners*, correlated poorly (*r* = 0.13, *p* > 0.05) [31]. Robust correlations between the qPCR and HTS approaches have also been observed in a non-microbial targeted investigation; a faecal-based dietary analysis of Little Penguins located in Western Australia revealed strong correlations (*r* ≥0.973) for four fish species [32]. In the analysis reported here, we found that some of the species-specific explorations illustrated that qPCR performance, and associated bacterial detection, was not as sensitive as that observed via HTS. This was most evident for bacterial species present in canine oral plaque at a lower relative abundance. These targeted taxa were determined to be absent from many samples analysed via the respective qPCR assay, but were detected at quantifiable levels upon assessment with HTS. Whilst qPCR assay design was based on a consensus sequence derived for each bacterial target, in this instance a similarity level of 99%, HTS amplification can be less specific and detect a broader range of related 16S targets. Optimization work with qPCR assay design could result in improved performance and increase the potential of these assays for utilization as diagnostic tools. As an example, this could look to extend the limit of detection (LOD) of each assay, given improved resolution and accuracy at the minimum threshold could enhance alignment with the respective HTS findings.

The popularity of machine learning approaches is increasing exponentially for numerous scientific applications [33–35], with algorithms that can enable more efficient routes to insights, and better decisions regarding best next steps. Here, using qPCR-derived data for three microbial biomarkers associated with canine periodontal health and disease, selected based on best overall statistical significance, prevalence, abundance and fold-change, we have utilised machine learning models to gauge sensitivity and specificity parameters. Similar performance from the five classification model types was found within each assay, suggesting stability of the model optimisation. For *Capnocytophaga* sp. COT-339, the KSVM machine learning model suggested 85.7% of periodontitis samples were correctly classified, whilst for non-periodontitis samples 27.5% were correctly classified. Based on the existing knowledge of a periodontal health association [6, 7, 36], the modelling outputs for this taxa do not necessarily align. As discussed above, this supports the perception that qPCR can be more specific than HTS, and in this instance, may have only been detecting a subset of the population of this bacterial taxa. Creating primers to a different region of the 16S rRNA gene may result in a broader spectrum assay leading to better amplification and therefore improved specificity. For Clostridiales sp. COT-028, the random forest machine learning model indicated that 60.0% of periodontitis samples were correctly identified, whilst the equivalent finding for Peptostreptococcaceae XI [G-4] sp. COT-019 was 74.3%. Given the disease association identified for both taxa [6, 7, 9, 11], it is encouraging that these probes correctly identify samples from dogs clinically diagnosed with periodontitis in around two thirds to three quarters of cases (60.0–74.3%). Non-periodontitis was correctly classified for 80.0% of clinically assessed samples with COT-028 and 67.5% of clinically assessed samples for COT-019, which is again encouraging that both assays detected a large proportion of the healthy cases. In the development of diagnostic tests, there can be a trade off in sensitivity and specificity, whereby a test may be good at confirming healthy subjects at the expense of potentially missing disease cases or, alternatively, good at diagnosing disease cases while erroneously describing healthy cases as diseased ones. The consequences of these scenarios need to be considered and the most appropriate balance determined for each type of diagnostic test.

Applied in conjunction with conscious plaque sampling and machine learning models, qPCR could therefore provide a tool to help resolve the significant prevalence verses diagnosis gap with canine periodontitis. We recognise that the current study has been conducted using a subgingival plaque sample set. However, an investigation comparing canine subgingival and gingival margin plaque has shown commonality in the microbiota observed across health and early periodontitis [9], thus supporting the employment of plaque from above the gum line, and hence conscious sampling, for microbial biomarker-based opportunities. Away from periodontal disease, numerous applications demonstrate the successful, practical employment of qPCR as a veterinary diagnostic tool. Prominent examples include zoonotic leptospirosis and leishmaniosis, caused by bacterial *Leptospira* spp., and protozoa *Leishmania infantum* and *L. donovani*, respectively [37–44]. Other canid relevant illustrations of the application of qPCR include the detection of bacterial *Ehrlichia* spp., for diagnosis of ehrlichiosis [45, 46], and atypical fungus *Pneumocystis*, a cause of *Pneumocystis* pneumonia in the immunocompromised [47, 48]. Such examples highlight true diagnostics. It is important to mention that while we use the term ‘diagnostic’, we appreciate that the qPCR assays provide new avenues to acquiring information on the presence of microbial taxa strongly associated with periodontal health or disease. As such, we envisage the qPCR tools developed to be utilised in numerous ways such as for health monitoring, disease staging and/or prognosis given the incredibly complex nature of the microbial communities associated with periodontal disease development and progression.

To-date, the most closely aligned work in the field to that described here has been performed by Kwon et al. [27], with qPCR-based detection of 11 human periodontopathic species, concluding *Treponema denticola*, as a possible prognostic biomarker for periodontitis in dogs. In the current study, the grouping of gingivitis (PD1) with healthy samples (PD0) for non-periodontitis was the same as the categorisation used by Kwon et al. [27] with the health status’ termed in tandem as the reversible group as opposed to the non-reversible group. There are other approaches which could be used for categorisation of samples; however, we believe this one has particular practical merit. Gingivitis (PD1) can be more readily identified upon visual, conscious assessment, whilst the diagnosis of periodontitis (PD2) typically requires a more detailed investigation, and represents the stage in the disease where a diagnostic test could potentially add most value.

Sample size is also a consideration and employing larger sample sets for each category used to build the models will enhance the quality and relevance of the outputs generated.

In addition, whilst we have applied machine learning methods to the qPCR data generated within this study to understand the diagnostic potential of single species taxa, similar algorithms could be applied to the existing HTS data to extract additional value from the historical work. Alternatively, accuracy parameters could also be estimated for combined multi-species models. Rapid-throughput laboratory approaches could then be employed to quantify multiple species in tandem via multiplex qPCR, with the ability to further refine and optimise diagnostic accuracy parameters. The KeyScreen™ GI Parasite PCR is a recent advancement and commercial example, enabling screening of 20 gastrointestinal parasites in domestic cats and dogs with greater sensitivity compared to traditional, microscopic centrifugal flotation detection methods [49].

The methods adopted within this study aim to demonstrate some of the initial steps that could be employed as part of a strategy to develop tools to support periodontal disease diagnosis. Molecular methods such as qPCR offer efficiency in many key areas of diagnostics development including cost, time and requirements for data processing and interpretation compared to other technologies such as HTS [50]. In turn, application of such an approach could be compatible with the capabilities of a commercial veterinary diagnostic service, with the finding and interpretation reported within 24–48 h post sample submission. Nevertheless, any outputs from machine learning models or laboratory-based assessments with promising diagnostic potential require testing in real world scenarios. Clinical validation studies, conducted with sufficiently large numbers of dogs across the full spectrum of periodontal disease (PD0-4), can confirm that predicted sensitivity and specificity parameters can be achieved.

## Conclusions

We report an approach which could be developed towards a qPCR-based diagnostic tool to detect microbial biomarkers of canine periodontitis in supragingival plaque. Based on strong correlations with HTS data, qPCR assays designed to target specific bacterial species offer an accurate, cost and time efficient strategy with promise for improving diagnosis of this prevalent yet under-reported condition.

## Methods

### Sample details

The sample set used for the study was generated in a previous study described by Davis et al. [6]. Briefly, subgingival plaque samples were harvested from a cohort of 223 dogs, which consisted of 72 with healthy gingiva (clinically normal; periodontitis stage 0, PD0), 77 with gingivitis (gingivitis only without attachment loss; periodontitis stage 1, PD1) and 74 with early periodontitis (attachment loss up to 25%; periodontitis stage 2, PD2), with the stage of periodontitis defined according to the American Veterinary Dental College (AVDC) [19] nomenclature. The subgingival plaque samples were pooled from multiple teeth of the same health state from the same dog. Specific details on these collections including inclusion/exclusion criteria, assignment of clinical health status and associated metadata can be found in Davis et al. [6]. Methods employed for DNA extraction are also available within Davis et al. [6]. Briefly, DNA was extracted from the subgingival plaque samples using the Epicentre Masterpure Gram Positive DNA Purification Kit, according to the manufacturer’s instructions with an additional overnight lysis. Extracted DNA was quantified and the purity determined using a NanoDrop ND1000 spectrophotometer (NanoDrop Technologies Inc).

### High-throughput sequencing (HTS) data

Plaque samples were analysed via 454 pyrosequencing to identify microbial taxa, see description in Davis et al. [6]. Taxonomy was assigned, and the number of sequence reads assigned to a particular taxonomic classification at both species and genus levels determined, as previously described by Davis et al. [6].

### QPCR assay development and validation

Proprietary qPCR assays were either designed in house or by Primer Design Ltd (Camberley, UK) based on 16S rRNA sequence information for individual taxa. Taxa selected for assay development were prioritised based on associations with periodontal health or disease, and rankings of presence (% of samples containing the species) and relative abundance (% of species within total bacterial population). All probes were designed with a fluorescein based (FAM) reporter dye, and TaqMan minor groove binder (MGB) or black hole quencher (BHQ), respectively. Briefly, in house assays were designed using full length consensus 16S sequences from a clone library, developed for a previous study to characterise canine oral microbiota [5]. Sequences were aligned in Vector NTI with the AlignX tool (Invitrogen™, Thermo Fisher Scientific Inc.) to enable regions of greatest variation to be identified around the 16S rRNA variable regions V1 and V2. Subsequently, Primer Express 3 (Applied Biosystems™, Thermo Fisher Scientific Inc.) was used to generate candidate primer and probe selections.

Performance of all assays was initially assessed using the 7900HT qPCR instrument (Applied Biosystems™, Thermo Fisher Scientific Inc.). Reactions mixes consisted of 5 µL Taqman Universal PCR Mastermix (2X) (Thermo Fisher Scsientific Inc. part no. 4,304,437), 0.5 µL primer and probe mix (20X), 1 µL template DNA and 3.5 µL nuclease-free water. Assay performance was quantified via reaction efficiency, a measure of the percentage of target molecules that are copied per PCR cycle, determined using a 10-fold dilution series of the amplified target clone DNA. For the purpose of this study, a cut-off of ≥80% efficiency was defined as a requirement for an assay to progress to subsequent phases of validation [51]. For determination of the limit of detection (LOD) and the limit of quantification (LOQ) additional 2-fold dilutions were added to the end of the dilution series. The LOQ was defined as the Ct for the lowest dilution where replicate test points were within 0.25 Cq of their median.

Assay specificity was assessed using the clone library with amplified clone DNA pooled into groups of 10 clones using equal amounts of DNA for each clone. Each assay was tested against all clone pools. Any clone pools not containing the target sequence but showing amplification were investigated further with each clone in the pool being tested individually. Cross reactivity against non-target clones was assessed based on the likely maximum proportion of the non-target clone, gauged from the 454 pyrosequencing data. For an assay to be accepted for further use, the contribution of the non-target signal, where the non-target sequence was present at its maximum likely proportion, could not exceed 10%.

### qPCR analysis of plaque DNA samples

The qPCR assays were performed on a subset of the same DNA samples, extracted from 205 of the subgingival plaque samples (70 with healthy gingiva, 69 with gingivitis and 66 with early periodontitis) used for HTS [6]. Briefly, qPCR reactions were conducted using the Biomark system with 48 × 48 assay chips (Standard BioTools Inc., previously Fluidigm Corporation Ltd). Due to the high throughput nature of the platform, a pre-amplification, enrichment step was conducted. A pre-amplification mixture was prepared, consisting of 25 µl TaqMan™ PreAmp Master Mix (Thermo Fisher Scientific Inc.), 12.5 µl pooled assay mix and 12.5 µl DNA. The pre-amplification conditions were an initial denaturation at 95 °C for 10 min, then 14 cycles with denaturation at 95 °C for 15 s and annealing at 60 °C for 4 min. The main qPCR amplification was then performed on the Biomark instrument, according to the manufacturer’s guidelines. Briefly, pre-amplified sample DNA, TaqMan™ Gene Expression Master Mix (Applied Biosystems™, Thermo Fisher Scientific Inc.) and sample loading reagent (Standard BioTools Inc.)), mixed with individual qPCR assays were combined for a total volume of 6 µL. Chips were primed, loaded and run according to the manufacturer’s instructions (Fluidigm® 48.48 Real-Time PCR Workflow Rev E) using the Integrated Fluidic Circuit controller and the Biomark instrument. Briefly, after priming the chip 20X mixtures of the primers and probes (Thermo Fisher Scientific Inc. or Primer Design) were combined with 2X Assay Loading Reagent (Standard BioTools Inc. part no. 85,000,736, previously Fluidigm Corporation Ltd) at a 1:1 ratio and loaded into the assay inlets on the chip. Taqman Universal PCR Mastermix (2X) (Thermo Fisher Scientific Inc. part no. 4,304,437) was mixed with 20X GE Sample Loading Reagent (Standard BioTools Inc. part no. 85,000,746, previously Fluidigm Corporation Ltd) and the sample DNA (or nuclease-free water for no-template controls) and loaded into the sample inlets on the chip. Positive controls consisted of a pooled clone DNA sample containing target sequences for all the qPCR assays. Thermal cycling was performed with a 95 °C for 10 min hot start phase followed by 30 cycles of 95 °C for 15 s then 60 °C for 1 min. Due to differences between the Biomark platform and the 7900HT system the LOQ cut-off for Biomark data was set at a Cq value of 21 for all assays.

### qPCR data analysis

Cq data were exported before analysis with GenEx™ v6.0 (MultiD Analyses AB, Sweden). The same thresholds were applied to all runs of the same qPCR assay across different Biomark chips. Briefly, outlying data points were removed using the outlier test included in GenEx™ which is based on Grubb’s test [52], with options to set the confidence level and cut-off standard deviation (SD). The confidence level was set to 0.10 and the SD cut-off level set to 0.01. Using the previously derived reaction efficiency levels (See Methods: qPCR assay development and validation) for each individual assay, adjustments were made to all data points to account for these differences. The mean was calculated for all replicate data points. All qPCR data were normalised to the level of a universal assay for each sample; this adjusted the data for differences in the overall amount of total bacterial DNA in each sample. This normalisation also enables for the presence of host DNA in the samples to be accounted for. The data were then linearised, such that the final qPCR data outputs were relative proportions (2^−(Cq.COT−Cq.Total)^). Samples with Cq.COT values outside of the reliable range of the assay (where Cq > 21) were assumed to have undetectable amounts of DNA and therefore those relative proportions were imputed as 0.

### Statistical analysis

#### Comparison of HTS and qPCR analysis technologies

Relative proportions for both HTS and qPCR outputs were log_10_ transformed (+ 0.0003 to allow for zeros, chosen for distribution and close to the minimum value) prior to analyses. Principal component analysis (PCA) was performed on the log_10_ proportions to assess the profile of the bacterial species and explore any potential clustering by analysis method or health state. Ellipses representing the 95% bivariate confidence region for PC1 and PC2 were calculated, assuming a multivariate t-distribution [53], for each analysis method and health state combination and included on the PCA score plot. Analyses were performed in R v4.2.1 statistical software [54], using the *vegan* [55] and *ggplot2* libraries.

The relative proportions of the HTS and qPCR analysis methods were then compared for each of the bacterial species by Pearson’s correlation coefficient. In addition, the non-parametric Spearman’s rank correlation coefficient was calculated to test the sensitivity of the correlation estimate to the imputed zero relative proportions in the qPCR assay due to the limit of detection.

#### Modelling for microbial diagnostic biomarkers

The qPCR data for each microbial taxa was modelled to evaluate single species prediction of periodontitis. Modelling was defined to classify between ‘periodontitis’ (PD2) or ‘not-periodontitis’ (health, PD0 and gingivitis, PD1) samples. Five classification machine learning methods were employed to estimate the diagnostic ability of each species: logistic regression (LR), linear discriminant analysis (LDA), random forest (RF), kernel support vector machines (KSVM) and weighted k-nearest neighbour (KKNN). The models were optimised to maximise the accuracy parameter (sum of correctly classified samples/total number of samples) using 5-fold cross-validation with hyper parameter tuning using a grid search.

Prior to modelling, the samples were split into a training and a test subset with stratification applied to the health state to reduce over specifying the ‘not-periodontitis’ state. Specifically, 72 samples (34 PD2: 18 PD1: 20 PD0) were used for training the models, and the remaining samples were used for bootstrap sampling the test subset, with 1000 repetitions in the ratio of 35 PD2: 20 PD1: 20 PD0 samples. The average accuracy, sensitivity (true positive rate: % of periodontitis samples correctly classified as periodontitis) and specificity (true negative rate: % of not-periodontitis samples correctly classified as not-periodontitis) and their standard deviations from those 1000 bootstrap repetitions were then used to estimate the performance of the models. The best performing models, out of the five approaches, were chosen to balance the associated risks associated with sensitivity and specificity. Averages are reported with standard deviation (SD).

The machine learning models were performed in R v4.2.1 statistical software, using the *mlr* [56] library.

### Electronic supplementary material

Below is the link to the electronic supplementary material.

Supplementary Material 1

## Data Availability

The datasets generated and/or analysed during the current study are not publicly available due to commercial reasons but are available from the corresponding author on reasonable request.
