# Supplementary material for: Developing diagnostic tools for canine periodontitis: combining molecular techniques and machine learning models
Source: BMC Vet Res. 2023 Sep 18;19:163. doi: 10.1186/s12917-023-03668-3 (PMC10507867; doi:10.1186/s12917-023-03668-3)
Supplement: Supplementary file 1 — Supplementary Material 1 [file 12917_2023_3668_MOESM1_ESM.docx]

**Supplementary Table 1. Pearson’s and Spearman’s Rank correlation coefficients between qPCR and HTS relative proportion data, with their associated p-values.**

|  | **Pearson's Correlation (r)** | | **Spearman's Rank Correlation (r_s_)** | |
| --- | --- | --- | --- | --- |
| **Truncated Assay Name** | **Coefficient** | **P-value** | **Coefficient** | **P-value** |
| COT-083 | 0.890 | <0.001 | 0.922 | <0.001 |
| COT-252 | 0.886 | <0.001 | 0.941 | <0.001 |
| COT-125 | 0.880 | <0.001 | 0.859 | <0.001 |
| COT-040 | 0.630 | <0.001 | 0.578 | <0.001 |
| COT-186 | 0.500 | <0.001 | 0.396 | <0.001 |
| COT-339 | 0.874 | <0.001 | 0.805 | <0.001 |
| COT-005 | 0.043 | 0.541 | -0.004 | 0.953 |
| COT-028 | 0.902 | <0.001 | 0.842 | <0.001 |
| COT-008 | 0.775 | <0.001 | 0.774 | <0.001 |
| COT-064 | 0.876 | <0.001 | 0.790 | <0.001 |
| COT-007 | 0.829 | <0.001 | 0.832 | <0.001 |
| COT-169 | 0.891 | <0.001 | 0.828 | <0.001 |
| COT-189 | 0.687 | <0.001 | 0.743 | <0.001 |
| COT-089 | 0.678 | <0.001 | 0.598 | <0.001 |
| COT-095 | 0.902 | <0.001 | 0.861 | <0.001 |
| COT-069 | 0.929 | <0.001 | 0.890 | <0.001 |
| COT-099 | 0.901 | <0.001 | 0.736 | <0.001 |
| COT-024 | 0.859 | <0.001 | 0.828 | <0.001 |
| COT-345 | 0.868 | <0.001 | 0.781 | <0.001 |
| COT-017 | 0.816 | <0.001 | 0.811 | <0.001 |
| COT-018 | 0.869 | <0.001 | 0.855 | <0.001 |
| COT-016 | 0.831 | <0.001 | 0.816 | <0.001 |
| COT-084 | 0.888 | <0.001 | 0.862 | <0.001 |
| COT-271 | 0.726 | <0.001 | 0.759 | <0.001 |
| COT-044 | 0.838 | <0.001 | 0.836 | <0.001 |
| COT-004 | 0.857 | <0.001 | 0.827 | <0.001 |
| COT-006 | 0.857 | <0.001 | 0.871 | <0.001 |
| COT-104 | 0.762 | <0.001 | 0.600 | <0.001 |
| COT-019 | 0.905 | <0.001 | 0.902 | <0.001 |
| COT-068 | 0.916 | <0.001 | 0.863 | <0.001 |
| COT-030 | 0.952 | <0.001 | 0.880 | <0.001 |
| COT-077 | 0.922 | <0.001 | 0.791 | <0.001 |
| COT-033 | 0.908 | <0.001 | 0.882 | <0.001 |
| COT-227 | 0.866 | <0.001 | 0.639 | <0.001 |
| COT-109 | 0.813 | <0.001 | 0.841 | <0.001 |
| COT-022 | 0.789 | <0.001 | 0.838 | <0.001 |
| COT-052 | 0.673 | <0.001 | 0.680 | <0.001 |
| COT-192 | 0.874 | <0.001 | 0.781 | <0.001 |
| COT-108 | 0.849 | <0.001 | 0.854 | <0.001 |
| COT-138 | 0.544 | <0.001 | 0.420 | <0.001 |
| COT-023 | 0.837 | <0.001 | 0.864 | <0.001 |
